# Wideband Dual-Polarized VHF Antenna for Space Observation Applications †

**DOI:** 10.3390/s20154351

**Published:** 2020-08-04

**Authors:** Alexandru Tatomirescu, Alina Badescu

**Affiliations:** Spl. Independentei, nr. 313, University POLITEHNICA of Bucharest, 060042 Bucharest, Romania; atatomirescu@munde.pub.ro

**Keywords:** wideband antenna, VHF, cross polarized, array element, radio detection, cosmic rays

## Abstract

This work presents the design for an antenna element that can be used in radio arrays for the monitoring and detecting of radio emissions from cosmic particles’ interactions in the atmosphere. For these applications, the pattern stability over frequency is the primary design goal. The proposed antenna has a high gain over a relative bandwidth of 88%, a beamwidth of 2.13 steradians, a small group delay variation and a very stable radiation pattern across the frequency bandwidth of 110 to 190 MHz. It is dual polarized and has a simple mechanical structure which is easy and inexpensive to manufacture. The measurements show that the ground has insignificant impact on the overall radiation pattern.

## 1. Introduction

The highest energy cosmic particles come from outside our solar system and their observation leads to a better understanding of the highest energetic processes across the Universe. Their detection is the subject of several radio experiments: CODALEMA in France [1], LOPES in Germany [2], LOFAR in Holland [3] and AERA in Argentina [4]. The work presented here is aimed to be useful for future planned experiments [5].

Depending on the cosmic particles’ energy level and mass, direct and indirect detection methods are used [6] for their detection. The indirect detection principle relies on the interactions that produce a broadband electromagnetic field in the radio frequencies. The radiation is coherent from frequencies ranging from a few MHz to up to few GHz [7]. The electromagnetic field is measured with networks of antennas and based on results the characteristics of the primary particle, which are retrieved with digital beamforming techniques [6]. In most applications, the VHF band is used because of the radio interferences in the UHF bandwidth.

The radio interferometer has specific requirements for the array element design. In order to have a wide scanning beam pattern, the array element has to have a low directivity and a wide beamwidth. At frequencies lower than 100 MHz, the natural cosmic background noise (manly coming from our galaxy) is high, thus, a high antenna gain/efficiency is not necessary [8]. Nevertheless, at higher detection frequencies, the antenna gain and bandwidth limit the sensitivity of measurements thus become a crucial part of the array’s design [6].

Given the number of elements in the array, the very large physical size of the network and the outdoor placement of antenna, the manufacturing and mechanical stability of each element is important and must be addressed early in the design process.

To limit the uncertainty in the reconstruction process, the array element pattern must be as stable as possible over observation angles and frequency band [6] (a stable antenna pattern facilitates the calibration procedure and the data post-processing). Each antenna should measure two orthogonal components of the electrical field [6], and this is why in this paper, a dual polarized antenna is addressed.

In previous experiments, different antenna designs were implemented and tested [6,9]. Among them, one worth mentioning is the short aperiodic loaded loop antenna (SALLA) [8] used in the Tunka–Rex experiment [10]. Although it is an inexpensive and easy-to-deploy antenna, the trade off is the narrow band pattern stability and the high dependence on the ground’s conductivity, which can vary significantly [7]. The same drawbacks appear in V-shape dipoles that were used in LOPES, LOFAR and CODALEMA experiments.

A Butterfly antenna design with a wide bandwidth and high stable gain was used in CODALEMA and AERA experiments. However, it is also affected by the ground conditions, which are highly whether dependent. Finally, the logarithmic periodic dipole antenna used in AERA (LPDA) has the advantages of both a wide bandwidth and a good gain. However, it is the most expensive to manufacture and deploy, and also susceptible at winds due to its height. In addition, it has a variable group delay that distorts the wideband cosmic ray pulse, which affects the reconstruction process.

An analysis of radio frequency interference [4] pointed out that the band of 110 to 190 MHz is relatively free of interferences and could replace or complement the already existing experiments below 100 MHz [6]. The work presented here addresses this design challenge. The solution has been chosen on the considerations of the largest possible bandwidth and stability of the radiation pattern over the band of interest for all observation angles. Practical consideration was given to the manufacturing cost and, more important, the ground reflections. Compared to other available models, the proposed array element has a stable radiation pattern and a low variation of the group delay over a larger relative bandwidth, but also a higher gain.

This paper is organized as follows: the next section briefly describes the design of the antenna (proposed and detailed in [11]) and introduces the new manufactured prototype. The following section presents the free-space antenna measurement method, the experimental results obtained (all in good agreement with simulations), together with the influence of the ground on the radiation pattern and the determination of group delay variations over incoming direction and frequencies. The last part summarizes the work and highlights the improvements compared to solutions adopted in other experiments.

## 2. Design Considerations and Prototype

The bow-tie antenna was chosen as a basic starting point for this work because of its mechanical simplicity. Such an antenna exhibits a good bandwidth and a wide beamwidth [6,12].

Due to the large physical size of the radiating elements (necessary at such frequencies) one of the main concerns was related to the high winds at which the antenna was exposed. A practical solution was the usage of aluminum mesh for the construction of the radiating elements. However, the mesh must be mounted on a solid metallic frame to prevent gravitational deformation and this could affect the radiation properties of the antenna. Another solution was designing the radiating elements as a grid of metallic bars. Both solutions had the disadvantage of a much higher manufacturing cost, especially when considering the large number of antennas required in arrays for radio detection of cosmic particles. Considering all these limitation, we preferred to use aluminum sheets for the radiating elements and position the antenna as close as possible to the ground to make it more resistant at high winds. A cut-off was inserted in the radiating elements to lower the wind resistance, to lower weight and also to limit the higher order modes.

**Figure 1:**
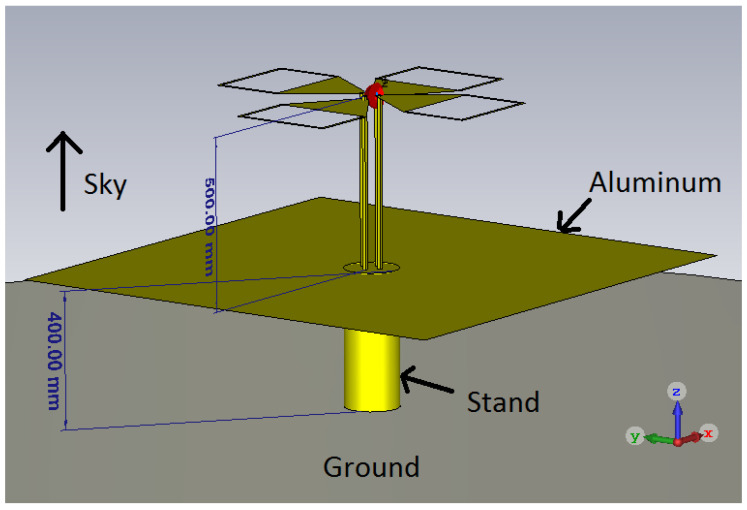
The simulated model of the proposed antenna, including the mounting on the ground. The feeding point is represented with red. The direction of the zenith is indicated [11] © 2018 IEEE.

**Figure 2:**
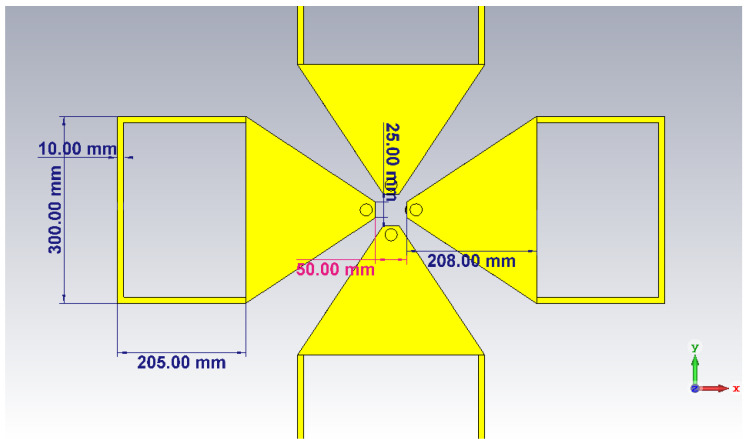
The dimensions of the radiating elements [11] © 2018 IEEE.

To avoid the effect of the ground reflection, a metallic plate was inserted between the radiating element and the actual ground, as illustrated in Figure 1. One of the dual polarized elements was oriented along the *x* axis (corresponding to the $\phi =0^{\circ }$) whereas the other element was oriented perpendicular, along the *y* axis (i.e., $\phi =90^{\circ }$). The zenith (i.e., $\theta =0^{\circ }$) is along the positive *z* axis. The exact dimensions of the radiating elements are shown in Figure 2 [11].

**Figure 3:**
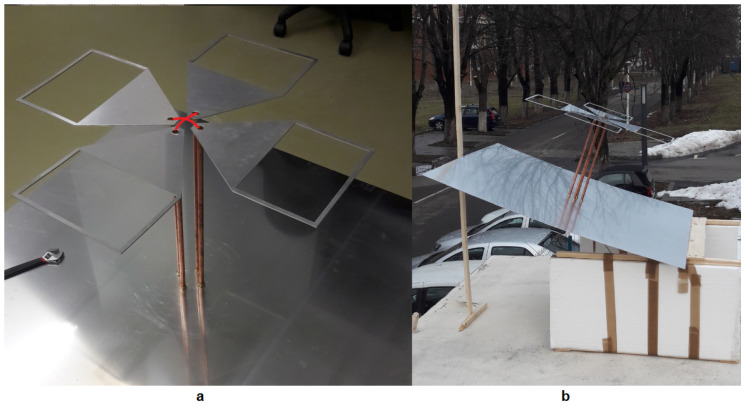
The antenna prototype: feeder (**a**) and a caption during measurements (**b**). The position of the feeding inner conductor of the coaxial cable is illustrated with red (**a**). The inner conductor is soldered on one blade and the outer conductor on the other, similar to [12] © 2012 IEEE.

The prototype is presented in Figure 3 and has an the overall height of less than 1 m. For the prototype, common construction materials were used: the radiating elements were plasma cut from a 2 mm thick aluminum sheet. The antenna was attached to the ground plate (a 3 mm thick aluminum square plate with a side of 1.4 m) using 15 mm diameter copper pipes and inserts. Holes in the ground plane and radiating elements were cut and threaded for easy assembly of the antenna. Usually, the dimensions of the metallic plate should exceed the dimensions of the antenna with λ/4 [13], however, large conductive plates also increase the edge effects. Decreasing the size of the metallic plate will degrade the peak gain at lowest frequencies and increase the back lobes towards the ground making it more susceptible to the conductivity of the ground. The final design represented the best compromise in terms of directivity pattern of the antenna and input reflection coefficient.

## 3. Results

### 3.1. Simulation Results

**Figure 4:**
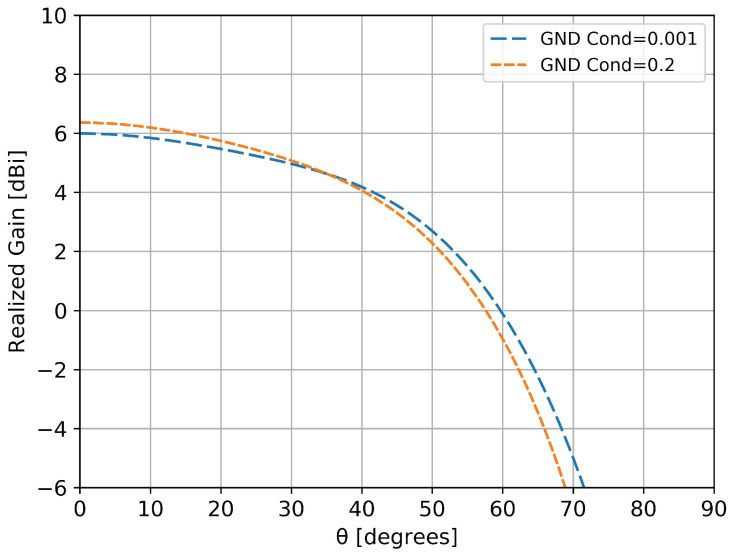
Variations in the elevation plane of the antenna situated on an infinite ground plane with different conductivities (σ=0.001 S/m is represented with blue line and σ=0.2 S/m with orange). Results are obtained at a frequency of 110 MHz, for an azimuth angle of $\phi =0^{\circ }$.

The numerical model of the antenna (with the metallic ground plane) was simulated with a commercial finite difference time domain electromagnetic solver, CST 2016. The first point of interest was the influence of the soil, so we performed simulations over an infinite ground plane with a finite conductivity in the 0.001 S/m–0.2 S/m interval [14] to cover different possible humidities of the soil. Figure 4 presents comparative results at 110 MHz for σ=0.001 S/m and σ=0.2 S/m. At higher frequencies, the effect of the ground was even smaller due to the larger electrical distance between the antenna and ground. It can be concluded that the influence of the soil’s conductivity on the radiation pattern is not higher than 0.3 dBm, thus, practically negligible.

**Figure 5:**
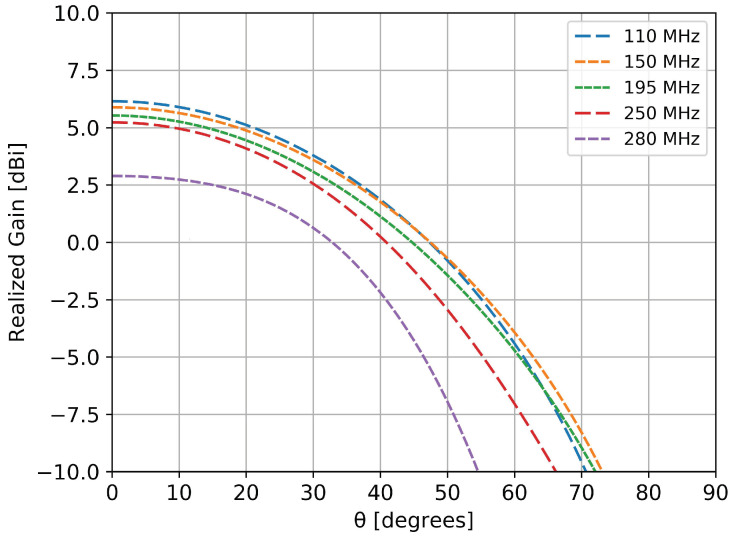
Radiation pattern in the elevation plane at different frequencies for the element along the *x* axis (cut in the $\phi =0^{\circ }$ plane).

**Figure 6:**
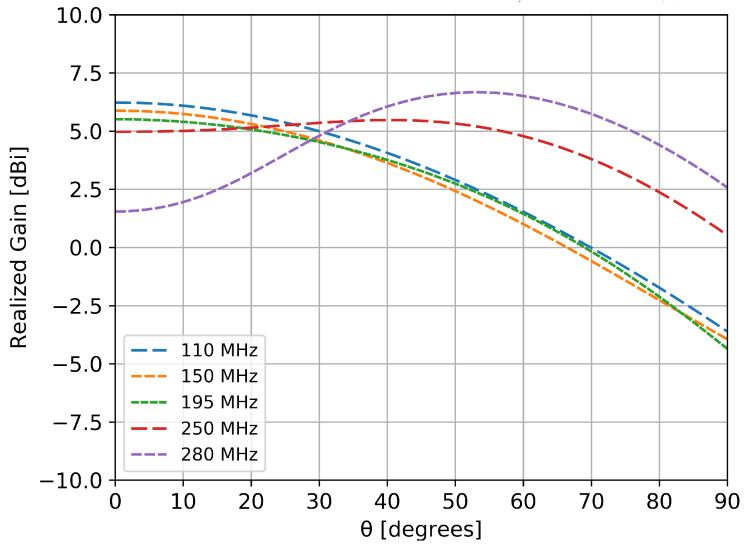
Radiation pattern in the elevation plane at different frequencies for the element along the *x* axis (cut in the $\phi =90^{\circ }$ plane).

As mentioned in the first section, a stable pattern over the frequency range of interest is mandatory for an accurate detection and estimation of the wideband pulse generated by the incoming cosmic particles. Figure 5 and Figure 6 show the variation of the gain with frequency in two azimuth perpendicular planes for the radiating element positioned along the *x* axis. Due to symmetry reasons, both elements (in *x* and *y* directions) had identical behavior.

Depending on the frequency of interest, the half power beamwidth in one plane was around $70^{\circ }$ (Figure 5) and around $100^{\circ }$ (Figure 6) in the other perpendicular one. An anti-resonance effect can be observed in Figure 6, around 300 MHz: the gain towards zenith is decreased due to the in-phase summation of the direct and reflected component by the metallic ground. However, in the 110–190 MHz band of interest the pattern is fairly stable, with variations of less than 1 dB.

In order to measure the incoming wideband field without distortions, the antenna should have a small variation of the group delay across the entire band of interest. The group delay itself is very important parameter as wavefront measurements totally relay on it.

**Figure 7:**
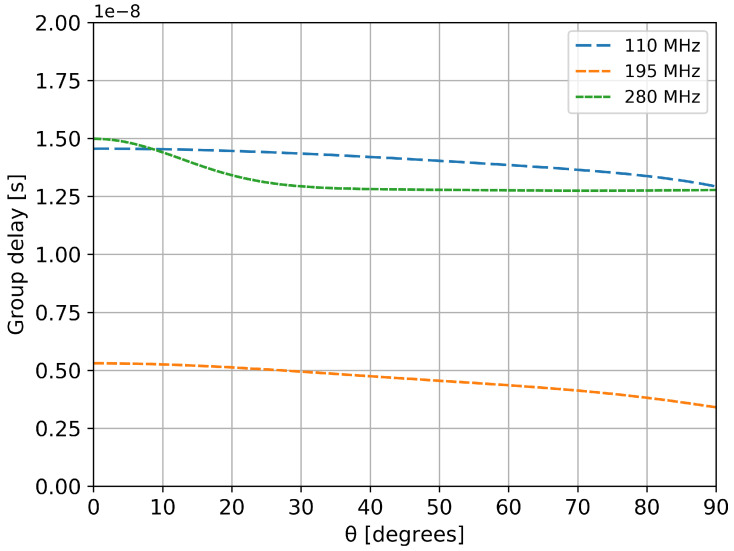
Simulated group delay variation in the elevation plane ( $\phi =0^{\circ }$).

The simulated group delay characteristic of the proposed antenna is presented in Figure 7 for various incoming zenith angles. The variation of the group delay is less than 2.5 ns at each frequency of simulation. This is a very important improvement compared to other experiments that shown a 80 ns variation [6]. Similar results are observed for other azimuth angles.

**Figure 8:**
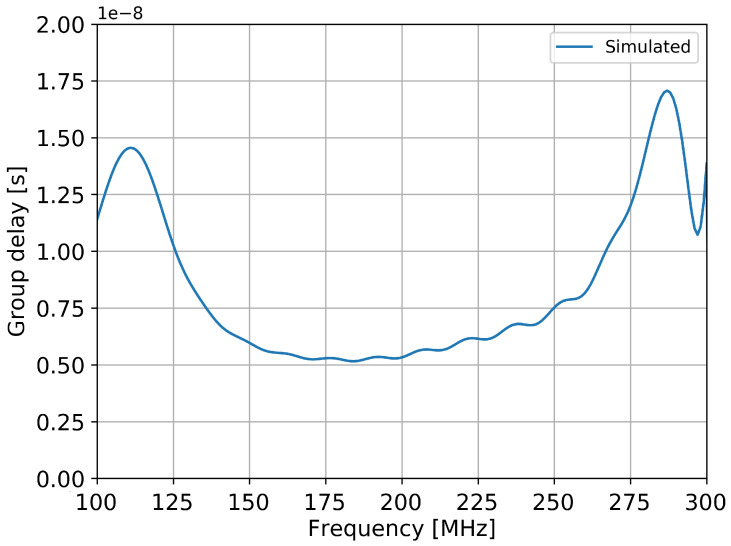
Simulated group delay variation with frequency for the zenith direction ( $\phi =0^{\circ }$).

The variation of the group delay with frequency is shown in Figure 8 (for the zenith incoming direction). Similar results are observed for other angles. Results from other experiments shown a variation of about 100 ns for Small Black Spider antennas, 25 ns for Butterfly antennas and about 10 ns for Salla antennas [9].

### 3.2. Experimental Results

Measurements of the radiation pattern were performed in an outdoor environment to meet the far-field conditions. This requirement arise due to the large dimensions of the antenna that would otherwise demand usage of a very large anechoic chamber.

The far-field slant elevated range method was adopted [15] to characterize the antenna. This method allows for transmission-loss free-space measurements of the gain product of two antennas, when the directive source is at the proper height. The accuracy is limited at ±0.35 dB [15]. The frequency range over which the method was proven to work is from 100 MHz to GHz, for various antenna types (simple dipoles, horns, etc.) [15].

**Figure 9:**
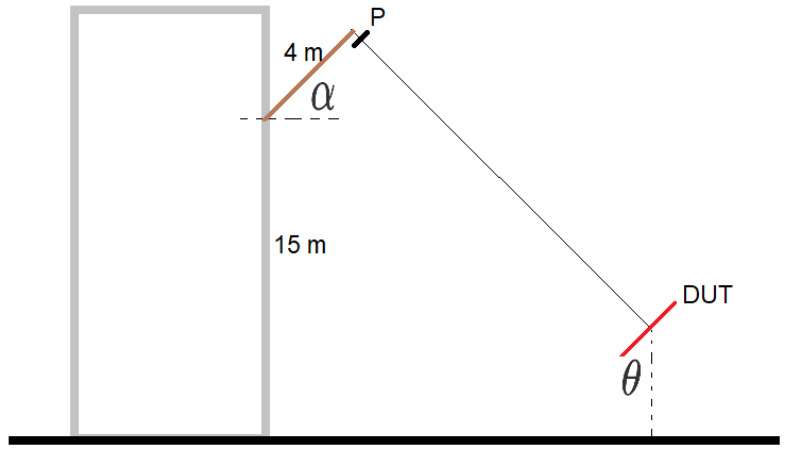
Slant elevated range principle (not at scale). The angle α is fixed at $45^{\circ }$. The inclination θ of the platform was varied where the device under test (DUT) was placed.

The measurement setup is presented in Figure 9. A calibrated emitting probe antenna (model BicoLOG 5070 produced by Aaronia) was fixed onto a 4 m pole outside the window of a building, approximately 15 m above ground. The pole was inclined at a fixed angle of $45^{\circ }$. The emitting antenna is represented as point P in Figure 9). The prototype (device under test, DUT, in Figure 9) was placed on an elevated platform (the elevated platform is also shown in Figure 2, right side). The DUT can be inclined at various θ angles values using a wooden support which allows for the determination of the elevation characteristics of the antenna. Both antennas were connected to a vector network analyzer (VNA) (from Deviser, model 7300 NA) that allows for the elimination of cable effects through calibration.

The realized gain $Gr_{DUT}\left(f\right)$ of the antenna under test can be calculated using Equation (1). It includes the mismatch losses, radiation losses and the directivity of the antenna compared to an isotropic radiator:

(1) $$Gr_{DUT}\left(f\right)=\frac{Gr_{Probe}\left(f\right)*H_{est}\left(f\right)}{S_{21}\left(f\right)}$$

where $Gr_{Probe}\left(f\right)$ is the realized gain of the antenna and is given by its manufacturer, $H_{est}\left(f\right)$ is the estimate of the channel using a reference antenna and $S_{21}\left(f\right)$ is the measured transfer parameter between the DUT and the probe antenna. The channel estimator was determined for a band of 400 MHz using calibrated antennas (for both emitter and receiver). A time domain gate was also applied [16] on the measured transfer parameter, and the method and results are carefully presented in [17]. The gating window introduces a 0.5 dB uncertainty [16].

**Figure 10:**
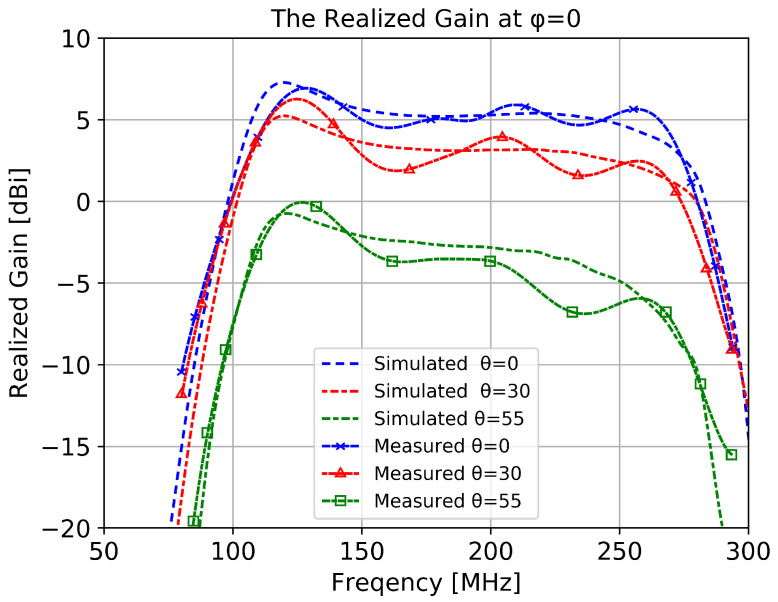
Simulated and measured radiation pattern versus frequency.

The prototype antenna was aligned to match polarization of the probe antenna and the DUT platform was inclined at three elevation angles using a wooden support. The results presented in Figure 10 show a good agreement with simulation (included for comparison). Even though low elevation directions show a lower gain, the gain is still higher than the usual requirements for such applications (i.e., −10 dB [6]). To eliminate the statistical errors (i.e., positioning errors and polarization mismatch loss), the measurements were repeated three times and the results in Figure 10 represent the mean values obtained.

**Figure 11:**
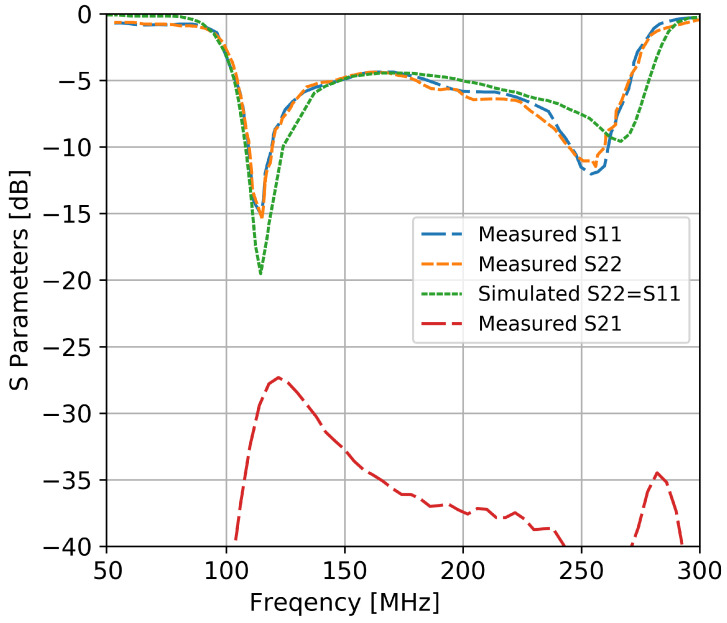
The simulated and measured S parameters of the prototype antenna.

The measured input reflection coefficients for the two perpendicular elements of the antenna are presented in Figure 11. Simulation results are also included in the plot for comparison. In the simulation, due to the symmetry of the structure, the two ports had a similar performance. The input reflection coefficient was smaller than −5 dB for the interval of 110 MHz to 190 MHz with a fractional impedance bandwidth of 88%. The elements also exhibit a good isolation between each other (i.e., higher than −27 dB).

**Figure 12:**
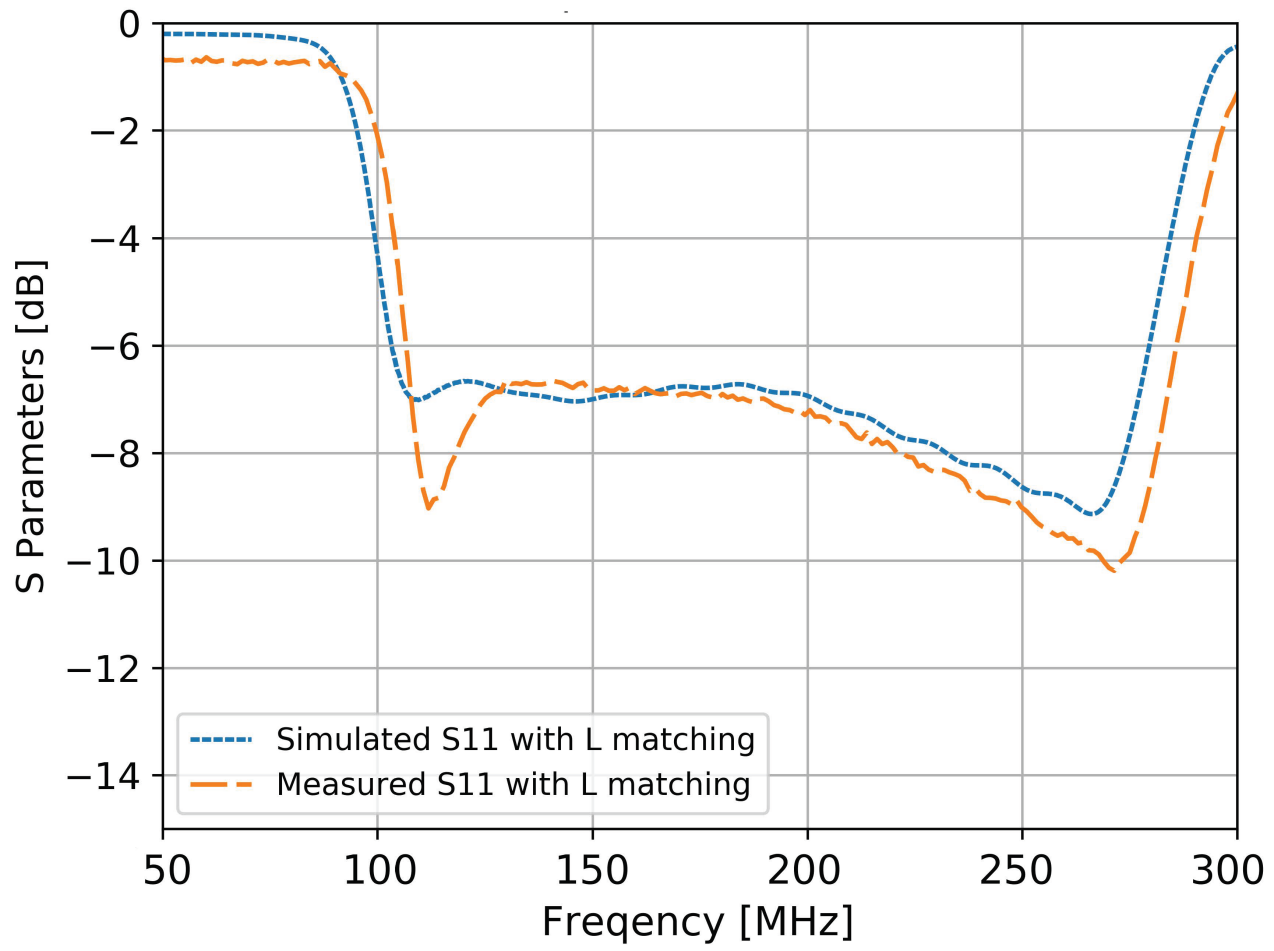
The simulated and measured S parameters of the antenna with a simple L matching network.

To increase the stability of the gain in the band of interest (necessary in the reconstruction process of particles’ characteristics) a simple L matching network was designed, consisting of a series lumped capacitor of 25 pF and a shunt wire inductor of 90 nH. An almost constant value of −7 dB is observed for the input reflection coefficient in Figure 12. The slight decrease at the frequencies around 300 MHz (i.e., outside the band of interest) is compensated by the decrease in gain thus a stable gain is acquired, without performance loss.

Around 300 MHz the field radiated by the bow-tie element and its reflection from the metal plate cancel each other forming an anti-resonance. The effect can be shifted to a higher frequency by lowering the element’s height.

## 4. Conclusions

In this work, we present the design of an antenna that can be used in an array configuration for detection and estimation of the electromagnetic radiation produced by cosmic particles that interact in the atmosphere. The main focus was on the 110–190 MHz frequency range, however, performances of the antenna were analyzed outside this regime for future possible extensions.

Low loss, lightweight, stable and cheap materials were used in the design, with low manufacturing complexity. The antenna has a small profile of less than a meter, so wind impact should be minimal. The influence from the ground was also minimal compared to other available solutions for similar applications (e.g., CODALEMA, AERA, etc.) [6].

The prototype has a very good bandwidth of 88% with a stable radiation pattern, with a good agreement between the simulations and measurement results. The performances in terms of the group delay are excellent, as the group delay is crucial for wavefront measurements and for the beamforming processes. Since the proposed prototype has a small Q factor, its group delay is small compared with the LPDA antennas used in previous experiments [6]. The variation of the group delay with frequency are much less than for Small Black Spider antennas and Butterfly antennas, and comparable with Salla antennas [9]. The disadvantage of the latter is a lower gain that increases the detection threshold in terms of cosmic-ray energy by about 30% [6].
